# Knowledge of Kratom among Alabama Pharmacists

**DOI:** 10.3390/pharmacy12010006

**Published:** 2023-12-30

**Authors:** Scott R. Penzak, Spencer H. Durham, Haley M. Phillippe, Brent I. Fox

**Affiliations:** 1 Department of Pharmacy Practice, Harrison College of Pharmacy, Auburn University, Auburn, AL 36849-5502, USA; durhash@auburn.edu (S.H.D.); mccrahl@auburn.edu (H.M.P.); 2 Department of Health Outcomes and Research Policy, Harrison College of Pharmacy, Auburn University, Auburn, AL 36849-5502, USA; foxbren@auburn.edu

**Keywords:** kratom, pharmacist, mitragynine, opioid, survey

## Abstract

Kratom (*Mitragyna speciosa*) is a botanical substance whose leaves produce stimulant- and opioid-like effects. Kratom use has increased precipitously in the United States (U.S.) over the last decade, yet, in our experience, many pharmacists are unfamiliar with this herb. The purpose of this study was to assess pharmacists’ awareness and knowledge of kratom. This cross-sectional study used an online questionnaire to preferentially solicit community pharmacists’ knowledge of kratom and collect demographic information. The survey was sent via email to approximately 10,000 pharmacists, targeting those in the state of Alabama, U.S. Data were analyzed using descriptive statistics, and the Chi Square test was used to compare nominal data. A total of 257 participants responded to the survey. Almost 50% of participants had heard of kratom, and 50% had not. Compared to females, males were more likely to have heard of kratom (64% vs. 42%; *p* = 0.0015), as were pharmacists who worked for an independent pharmacy vs. a chain (61% vs. 41%; *p* = 0.025). Of the participants who had heard of kratom, only 14% considered themselves knowledgeable or very knowledgeable about the herb, and only 44% knew it was illegal in Alabama. These data indicate a need to further kratom education among community pharmacists in Alabama.

## 1. Introduction

Mitragyna speciosa (*Rubiaceae*) is a tropical tree indigenous to parts of Africa and Southeast Asia [1,2]. The tree is commonly known as “kratom”. Kratom leaves are harvested from the tree, dried, and consumed as a tea or in its raw form [3]. Kratom may be purchased as whole leaves, in powder form, as encapsulated powder, or as an encapsulated or liquid extract. When sold to the public, kratom is categorized by its color and strain [3,4,5]. The color of kratom differs, as does its alkaloid content, based on the age of the plant. In its younger form, kratom is referred to as white vein kratom [3]. White vein kratom is promoted as providing energy, increasing mental focus, and lifting mood [3,4,5]. Green vein kratom is represented by the maturing plant and is touted as producing euphoria, positivity, and talkativeness [3,4,5]. Finally, red vein kratom represents the fully matured plant; it is advertised as being mildly sedating, relaxing, and calming [3,4,5]. Within these three kratom veins exist many different strains, which include Maeng Da, Sumatra, Malay, and others [3,4,5]; these strains are usually indicative of where the kratom is grown (i.e., Thailand, Island of Sumatra, and Malaysia, respectively). Due to differences in climate and growing conditions, different kratom strains may contain varying amounts of alkaloids, which can produce diverse pharmacologic effects [3]. Kratom has been used for centuries in indigenous areas to treat anxiety; provide energy; and relieve pain, diarrhea, cough, and depression [6,7]. It is part of the culture and tradition in some geographical regions, such as the southern peninsula of Thailand [3,6].

Although it cannot be advertised in the U.S. to treat any medical condition, kratom is frequently used as an opioid substitute, to treat opiate withdrawal, enhance mood, and control pain [8,9,10]. The pharmacologic effects of kratom are believed to be due to the presence of alkaloids, over 40 of which have been isolated from samples of the herb [11,12]. The two primary active ingredients in kratom are the indole alkaloids mitragynine (MG) and 7-hydroxymitragynine (7-OH-MG), which make up ~60% and ~2% of kratom’s alkaloid content, respectively [13,14,15]. Both MG and 7-OH-MG bind to the mu opioid receptor [16]. MG is also capable of binding to the alpha-2 adrenergic, dopamine D_2_, serotonin, and adenosine receptors, although these interactions are not well described [13,17]. In a series of in vitro experiments, Gutridge et al. showed that in addition to binding to the mu opioid receptor, 7-OH-MG acts as a competitive antagonist at kappa and delta opioid receptors [16]. In preclinical experiments, MG was observed to be 5–10 times less potent than morphine [18]. In other preclinical investigations, 7-OH-MG was reported as being 46 times more potent than MG and 13 times more potent than morphine at the mu opioid receptor [15,19]. Lower doses of kratom (1–5 g of the raw leaves) produce mild central nervous system stimulation, while higher doses (5–15 g) produce classic opioid effects such as euphoria, analgesia, and relief of opioid withdrawal symptoms [7,10,20].

Over the last decade, kratom use has expanded overseas and is now available in the U.S. As of 2020, several surveys in the U.S. estimated that 0.7% to 0.8% of adults had taken kratom at some point, and the lifetime prevalence of kratom consumption was reported at 1.3% [21,22]. Potential reasons for increasing kratom use in the U.S. are (1) ease of accessibility via “smoke shops”, gas stations, and online vendors and (2) fewer traditional opioid-related side effects, such as respiratory depression, that are typically associated with opioid medications [3,14]. The most common side effects reported with kratom, in the absence of concomitant drugs of abuse, are minor and include hypertension, confusion, vomiting, drowsiness/lethargy, nausea, tachycardia, and agitation/irritability [23]. Despite what appears to be a generally favorable safety profile, cases of kratom toxicity, including fatalities, have been reported. In 2019, the U.S. National Poison Data System reported receiving 885 calls where kratom was the sole agent of exposure, compared to only 18 such calls in 2011 [24,25]. Of these 885 calls, 78 cases were associated with major adverse events, including three fatalities. In addition to toxicities, another important risk associated with kratom use is tolerance, physical dependence, and withdrawal symptoms upon abrupt cessation [26].

Because kratom is considered a supplement and not a drug, it is not subject to regulatory oversight by the U.S. Food and Drug Administration (FDA). Kratom is legal in the United States except in Alabama, Arkansas, Indiana, Tennessee, Vermont, and Wisconsin [19,26]. Other states or cities, such as New York and New Jersey have restrictions on the sale or use of kratom [27,28]. The U.S. Food and Drug Administration continues to express concern over kratom’s safety and abuse potential [27,29]. From a global perspective, kratom is illegal in Australia, Thailand, Malaysia, and Myanmar, and it is designated as a controlled substance, or regulated in some manner, in Finland, Denmark, Sweden, Romania, Lithuania, Poland, Germany, Ireland, and New Zealand [30,31]. In many areas of the world, kratom’s legal status remains unstable and subject to change.

Despite the increasing use/abuse of kratom in the U.S. and publicity surrounding its legal status, we hypothesized that many pharmacists are not familiar with this botanical substance and the risks associated with its use. The purpose of this study was to survey pharmacists on their knowledge and awareness of kratom. Community pharmacists in the state of Alabama were targeted. Demographic information was collected to identify whether specific characteristics (i.e., country of origin, sex, years in practice, etc.) were associated with knowledge of kratom. The primary aim of this investigation was to assess whether there is a knowledge deficit with regard to kratom among community pharmacists in Alabama.

## 2. Materials and Methods

This study was designed as a prospective cross-sectional survey (Qualtrics, Provo, UT, USA) among pharmacists licensed in Alabama, U.S. The survey questionnaire was distributed to community pharmacists to assess their knowledge of kratom and collect their demographic information. Subject participation in this study was voluntary and anonymous. No incentives were provided for participation.

### 2.1. Study Participants

Participants were self-identified community pharmacists practicing in the state of Alabama whose email addresses were part of a continuing education listserv kept by the Division of Clinical Affairs and Outreach within the Harrison College of Pharmacy (HCOP) at Auburn University, Auburn, Alabama. Participants had internet access and the ability to fill out the survey.

### 2.2. Questionnaire

A self-administered online questionnaire was used for data collection. The questionnaire contained 19 questions (Appendix A). The first 8 questions collected pertinent demographic information. The remaining 12 questions assessed participants’ knowledge of kratom, including information such as purported indications, pharmacologic effects, and legal issues surrounding its use and availability.

### 2.3. Data Collection

The questionnaire was initially piloted by the study investigators and a group of experts and assessed for face validity using the methodology described by Hardesty and Bearden [32]. This resulted in minor revisions to improve clarity. The Auburn University Institutional Review Board approved the finalized survey, and all research was conducted according to the World Medical Association Declaration of Helsinki. This survey was sent via email to approximately 10,000 self-identified community pharmacists practicing in the state of Alabama using a Qualtrics survey described above. All participants provided electronic informed consent.

### 2.4. Data Analysis

Data were imported and arranged in Microsoft Excel (Microsoft Corp, 2016, version 16.0.5422.1000), Redmond, WA, US) (accessed on 1 August 2023). Quantitative demographic data were summarized using descriptive statistics and reported as frequencies and percentages. Inferential statistical tests were used to compare demographic differences between those community pharmacists who had heard of kratom and those who had not. Due to the nominal nature of the data, the chi-square test was used to analyze significant differences between these two groups. *p* < 0.05 was accepted as statistically significant. For 2 × 2 contingency tables, Yates correction was applied to the Chi-square statistic and subsequent *p* value (chi-square test calculator [https://www.socscistatistics.com/tests/chisquare2/default2.aspx]) (accessed on 5 August 2023).

## 3. Results

### 3.1. Participant Demographics

A total of 257 responses were included in the final analysis. The demographic details of the participants are presented in Table 1.

The majority of study participants were 26–45 yrs (56%) or older than 56 yrs (28%). A minority of respondents were between 46 and 55 yrs (15.5%), and one was less than 25 yrs. Almost two-thirds of the participants were female (~65%) and nearly a third were male (~35%). The majority of participants had a Pharm.D. (Doctor of Pharmacy) degree as their sole pharmacy degree (~61%), while nearly a third (~34%) had a Bachelor of Science (BS) degree in Pharmacy as their sole pharmacy degree. The remainder of the participants (5.5%, collectively) had a combination of degrees. Most participants (~85%) reported not completing any post-graduate training programs. Over 90% of participants had been in practice for more than four years and over a third (~36%) had been in practice for greater than 21 years. The overwhelming majority of participants were raised (“grew up”) in North America (~98%) and practice in Southeast Alabama (~89%).

### 3.2. Knowledge of Kratom

The number of participants who had previously heard of kratom was 50.2% compared to 49.8% who had not. Table 2 lists and compares covariates between these two groups.

Participants who had previously heard of kratom were more likely to be male vs. female (64% vs. 42%; *p* = 0.0015) and to work in an independent pharmacy vs. a chain (61% vs. 41%; *p* = 0.025). No other demographic variables differed significantly between participants who had/had not heard of kratom. These included age, terminal degree, geographic location of origin (e.g., where the individual was raised), level of post-graduate training, years as a practicing pharmacist, and practice location within Alabama (*p* > 0.05 for all comparisons).

Of the 50.2% of participants who had heard of kratom, 14% considered themselves knowledgeable or very knowledgeable about the herb, 30% expressed confidence in understanding why people take kratom, and 19% were confident in their ability to explain the clinical effects of kratom. Only 44% of respondents who had heard of kratom knew it was illegal in Alabama, and only 16% felt confident that they could discuss the legal issues surrounding the herb.

Nearly a third (31.3%) of participants who had heard of kratom reported that a patient had asked them a question about it. Notably, 68% of respondents who had heard of kratom indicated that they had read a lay-press article about it compared to 27% who had read a scientific article about it (*p* < 0.00001). Interestingly, seven participants (5.43%) who had not heard of kratom indicated that they had heard of mitragynine and/or 7-hydroxymitragynine. Of those who had heard of kratom, 18.8% reported having heard of either mitragynine and/or 7-hydroxymitragynine.

## 4. Discussion

This study aimed to evaluate kratom knowledge among community pharmacists in Alabama, U.S. Despite a seemingly large body of mainstream information on kratom, numerous scientific reviews of its efficacy and safety, and well-publicized legal issues, only half of the Alabama community pharmacists we surveyed had heard of kratom, and even fewer were aware it was illegal in the state in which they practiced [3,19,27,29,33]. While we hypothesized that not all pharmacists would be familiar with kratom, the magnitude of these results was still somewhat unexpected (i.e., that only half of surveyed pharmacists had heard of kratom), considering state-wide publicity in May of 2016 when Alabama legislators passed Senate Bill 226, which made possession and/or sale of kratom illegal [34]. This was several months before the U.S. Drug Enforcement Administration (DEA) identified kratom as a “drug of concern” and planned to make it a Schedule I medication before ultimately reversing that decision and keeping it legal at the federal level [35,36]. This magnitude of statewide and national publicity might have been expected to increase kratom awareness among Alabama community pharmacists. On the other hand, because kratom is illegal in Alabama and is not available in pharmacies, pharmacists may be less cognizant of its existence. As noted earlier, kratom is also illegal in Arkansas, Indiana, Tennessee, Vermont, and Wisconsin [28]. It would be interesting to investigate how pharmacist knowledge of kratom in these states compares with kratom knowledge among pharmacists in Alabama. If it is comparatively limited, collaborative efforts between the states could be undertaken to educate pharmacists practicing in these locations; this could take the form of continuing education articles, presentations, or symposia. However, it is possible that the legal status of kratom has no bearing on pharmacist knowledge of kratom. Comparative studies of kratom knowledge among pharmacists in states where kratom is and is not legal would address this question.

In addition to the legal status of kratom, another variable that may impact a pharmacist’s knowledge of kratom is whether that individual learned about it during the course of their pharmacy education. There are two pharmacy schools in the state of Alabama: Auburn University’s Harrison College of Pharmacy (HCOP) and Samford University’s McWhorter School of Pharmacy. Neither program routinely includes kratom as a core component of their respective curriculums. Some students may encounter kratom during Advanced Pharmacy Practice Experiences (APPE). However, this is likely “hit or miss”, with some students learning about kratom and others not encountering it. Given the relatively recent increase in kratom availability and use in the United States, one might expect that recent graduates would be more likely to be familiar with kratom; however, our data did not show this to be the case, as the percentage of pharmacists who had heard of kratom who had been practicing for 0–6 yrs, 7–20 yrs, and >21 yrs were 52%, 43%, and 55%, respectively (*p* = 0.276).

Since the premise of our study hinges on pharmacist knowledge of kratom, it begs the question, “Why is it important that pharmacists possess a basic understanding of kratom pharmacology and toxicology?” The answer is, “To be able to provide accurate information to individuals who may be using or considering using kratom”. Due to their accessibility to the public, pharmacists are likely to represent the first team of healthcare professionals that consumers will seek if they have questions about kratom. If a pharmacist is unable to provide a patient with accurate information about kratom, that individual is likely to search the internet for answers to their questions. While the internet can often serve as an outstanding data source on a variety of topics, it can also provide inaccurate and/or misleading information. Websites that sell kratom tend to list benefits of the herb without providing risks and potential side effects. Pharmacists are in a unique position to be able to offer inquiring individuals with balanced and correct information about kratom, including lack of standardized product strengths, so they can make informed decisions regarding its use. For example, certain kratom products consisting of potent mitragynine extracts that are much stronger than kratom powder and carry additional risks. This type of information may not be easily extricated from online articles by would-be kratom users who are not medically trained.

A limitation of our study is that we simply asked participants whether they had been asked a question about kratom from one of their customers. We did not ask about the nature of said question, and whether it related to kratom’s putative benefits, toxicities, or potential to produce physical dependence and withdrawal. Although kratom has been touted as a less addictive alternative to prescription opioids and illicit opioids (i.e., heroin), there is a growing body of evidence that regular kratom use can result in both physical and psychological dependence and addiction [37,38]. Several studies in rodents reported that abrupt cessation of MG can produce withdrawal symptoms within 24 h of the last dose [39,40,41]. Additional studies in humans and case reports have shown that kratom can produce physical and psychological dependence [37]. The most frequently reported symptoms of kratom-associated withdrawal include sleeping difficulty, decreased appetite, nausea, vomiting, muscle spasms, sweating, fever, abdominal pain, diarrhea, headaches, hot flashes, watery eyes, hiccups, tremors, body aches, severe muscle pain, and cramps [1,37]. It is important that pharmacists be familiar with this constellation of symptoms so they can raise awareness among patients. Further, it is important that pharmacists understand that kratom withdrawal may be debilitating for some patients, and warn them accordingly when asked about risks associated with kratom use.

Most kratom users in the U.S. are male (56.9%) and between the ages of 31 and 50 years [38]. Interestingly, among our survey respondents, male pharmacists were significantly more likely to have heard of kratom (*p* = 0.0015). This may arise from male kratom users feeling more comfortable discussing their kratom use with male pharmacists, thereby informing the latter of this botanical substance. Nevertheless, this is speculative, as only 31% of participants who had heard of kratom had been asked about it by a customer. Survey respondents who worked in an independent pharmacy were significantly more likely to have heard of kratom than those who worked for a chain drugstore, regardless of the chain size. Since some pharmacists presumably learn of kratom through conversations with their patients, pharmacists practicing at independent pharmacies may be more likely to hear about kratom, given that personalized customer contact tends to be emphasized more in that setting [42].

Another demographic factor we assessed for its potential impact on kratom familiarity was the geographic location in which the surveyed pharmacist was raised. We anticipated that pharmacists originally from areas where kratom is indigenous (i.e., Thailand, Malaysia, Myanmar, and other areas of Southeast Asia) would be more likely to be familiar with it. However, since 98.4% of survey participants grew up in North America, it was not possible to make this assessment. Similarly, we postulated that pharmacists practicing near the borders of Alabama’s four surrounding states (Mississippi, Tennessee, Georgia, and Florida) would be more inclined to have heard of kratom since it is legal in all four of those territories [42]. However, the majority (89.4%) of survey participants practiced in southeast Alabama, where Auburn University’s Harrison College of Pharmacy is located. Thus, the greater response rate among community pharmacists located in this area might have been because the survey invitation originated from an Auburn University email address. The remaining ~10% of respondents were scattered throughout the remainder of the state. As a result, neither geographical origin nor practice location within Alabama were able to shed light on which community pharmacists were more likely to have heard of kratom.

Interestingly, participants who had heard of kratom were more likely to have read a lay-press article about it than a peer-reviewed scientific publication (68.0% vs. 27.3%, *p* < 0.00001). These data suggest that study participants were more likely to find information on kratom in a lay-press article than in educational materials such as pharmacy journals and/or continuing education programs. This is concerning, since articles in the lay press are not known for their scientific rigor, are not peer-reviewed, and may contain biases. This is especially true for kratom, given the prevalence of highly charged opinions surrounding its safety, efficacy, and legal status [3,19,35,36]. These results highlight the need for further sharing of information, perhaps ideally in the pharmacy literature, to educate community pharmacists in Alabama about kratom. However, this should not be construed to imply that there is a paucity of peer-reviewed information on kratom in the pharmacy literature. While a detailed report of all kratom-related publications in the pharmacy literature is beyond the scope of this discussion, a detailed pharmacologic and clinical assessment of kratom was published by White et al. in the American Journal of Health-System Pharmacy in 2019 [19]. Also in 2019, Eggleston et al. published a review on kratom use and toxicities in the United States in Pharmacotherapy [24]. While both of these journals are well-respected among the pharmacy profession, they may appeal more so to clinical and in-patient pharmacists and less so to pharmacists practicing in a community setting. There is a single case of hepatomegaly associated with kratom use published in the Journal of the American Pharmacy Association [43]. A comprehensive assessment of pharmacy journals, and whether they have addressed kratom use, may be useful in identifying opportunities to expose more community pharmacists to kratom, its pharmacology, toxicity, and use patterns.

A limitation of this study is that our survey targeted community pharmacists. It would be interesting to see whether pharmacists practicing in other areas, such as in- and out-patient clinical pharmacists and dispensing hospital pharmacists, are more or less informed than community pharmacists regarding kratom. It would also be useful to learn how other clinicians, such as physicians, physician assistants, and nurse practitioners fare regarding their knowledge of kratom. The authors’ anecdotal experience (i.e., routine daily interactions) suggests that these practitioners may be even less familiar with kratom than community pharmacists; prospective investigations are needed to either confirm or refute this hypothesis.

A further consideration is the relatively low response rate to the survey (257 replies from approximately 10,000 pharmacists). While we attempted to design our survey in a manner that would maximize responses (i.e., concise, focused questions that could all be answered in less than 12 min), the fact that we did not offer an incentive and general survey fatigue among professionals likely contributed to the number of responses we received. In spite of this response rate, we still believe that the conclusions drawn from this sample are a valid representation of the population.

Kratom use has become increasingly common in the U.S. and there are disagreements among users, regulators, scientists, and clinicians regarding its safety, efficacy, and legality [3,19,35,36]. Despite the increasing visibility of this botanical substance, and recent publicity surrounding its legal status, only 50% of community pharmacists in Alabama, U.S., are familiar with kratom, and even fewer are aware of its legal status in the state in which they practice. The current investigation highlights the importance of developing educational materials to improve kratom awareness among community pharmacists, thereby paving the way for pharmacists and other healthcare professionals to provide optimal care for patients where kratom is concerned. 

## Figures and Tables

**Table 1:** Demographic characteristics.

Characteristics	N	%
Gender		
Male	91	35.4
Female	166	64.6
Age (yrs)		
<25	1	0.39
26–35	73	28.4
36–45	71	27.6
46–55	40	15.6
56 and above	72	28.0
Terminal degree		
Bachelor of Science in pharmacy only	87	33.9
Bachelor of Science in pharmacy plus a non-Pharm.D. degree	6	2.3
Bachelor of Science in pharmacy plus Pharm.D. degree	5	2.0
Pharm.D. plus a Master of Business Administration degree	3	1.2
Pharm.D. as sole degree	156	60.7
Geographical location of origin		
North America	253	98.4
Other	4	1.6
Level of post-graduate (PG) training		
PGY1 ^a^ general residency	8	3.1
PGY1 ^a^ community residency	10	3.8
PGY2 ^b^ community residency	2	0.77
Fellowship	3	1.2
Other	16	6.1
None	222	85.1
Place of employment		
Independent pharmacy (less than 4 stores)	89	34.6
Small chain pharmacy (5–1000 stores) ^c^	13	5.1
Intermediate chain pharmacy (1001–5000 stores) ^d^	23	9.0
Large chain pharmacy (more than 5000 stores) ^e^	73	28.4
Other	57	22.2
Years practicing pharmacy		
0–3	19	7.4
4–6	46	17.9
7–10	33	12.8
11–20	59	23.0
More than 21	93	36.2
Practice location in Alabama	4	1.6
Midwest	9	3.5
Northeast	2	1.2
Southeast	230	89.5
Southwest	5	2.0
Other	3	1.2

^a^ Post-graduate year 1; ^b^ post-graduate year 2; ^c^ examples: Costco, Kaiser Permanente, and Cardinal Health; ^d^ examples: Publix, Albertsons, Kroger, Rite AID; ^e^ examples: CVS, Walgreens.

**Table 2:** Comparisons between those who have and have not heard of kratom.

Comparators	Heard of Kratom (%) ^a^	*p* Value
Gender		
Male	63.7	0.0015 *
Female	42.1	
Age (yrs)		
26–35	43.8	0.498
36–45	47.9	
46–55	50.0	
56 and above	56.9	
Terminal degree		
Bachelor of Science in Pharmacy only	52.9	0.479
Bachelor of Science in Pharmacy plus a non-Pharm.D. degree	33.0	
Bachelor of Science in Pharmacy plus Pharm.D.	80.0	
Pharm.D. plus a Master of Business Administration degree	66.7	
Pharm.D. as sole degree	47.4	
Geographical location of origin		
North America	49.8	0.994
Other	50.0	
Level of post-graduate (PG) training		
PGY1a general residency	50.0	0.105
PGY1a community residency	90.0	
PGY2b community residency	33.3	
Fellowship	66.7	
None	47.3	
Place of employment		
Independent pharmacy	60.7	0.025 *
Chain pharmacy	41.3	
Other	50.9	
Years practicing pharmacy		
0–3	52.6	0.460
4–6	52.1	
7–10	36.4	
11–20	47.4	
More than 21 years	54.8	
Practice location in Alabama		
Midwest	75.0	0.207
Northeast	22.2	
Northwest	33.3	
Southeast	49.6	
Southwest	80.0	
Other	66.7	

^a^ The percentage is that of each demographic variable. For example, 58 of 91 males (63.7%) reported having heard of kratom, compared to 70 of 166 females (42.2%) who reported having heard of kratom (*p* = 0.0015).* Denotes statistical significance

## Data Availability

The data presented in this study are available in the included tables.

## Appendix A. Survey of Kratom Knowledge and Participant Demographics

Q1 Age:
❍ <25 yrs (1) ❍ 26–35 yrs (2) ❍ 36–45 yrs (3) ❍ 46–55 yrs (4) ❍ 56 yrs (5)
Q2 Gender:
❍ Male (1) ❍ Female (2)
Q3 Professional degree:
❍ DO (1) ❍ MD (2) ❍ Pharm.D. (3) ❍ BS in pharmacy (4) ❍ Other (5) ____________________
Q4 In what geographical area did you grow up?
❍ North America (1) ❍ South America (2) ❍ Europe (3) ❍ Southeast Asia (4) ❍ Other (5) ____________________
Q5 Please indicate your level of post-graduate training (please select all that apply)?
❍ PGY1 general residency (1) ❍ PGY1 community residency (2) ❍ Fellowship (3) ❍ Other (4) ❍ None (5)
Q6 How would you describe the pharmacy in which you currently work?
❍ Independent pharmacy (less than 4 stores) (1) ❍ Small chain pharmacy (5–1000 stores) (2) ❍ Intermediate chain pharmacy (1001–5000 stores) (3) ❍ Large chain pharmacy (more than 5000 stores) (4) ❍ Other (5)
Q7 How many years have you been practicing independently (i.e., since you completed post-graduate training if applicable)
❍ 0–3 years (1) ❍ 4–6 years (2) ❍ 7–10 years (3) ❍ 11–20 years (4) ❍ 21 years or more (5)
Q8 What geographical region is your work institution?
❍ Midwest (1) ❍ Northeast (2) ❍ Northwest (3) ❍ Southeast (4) ❍ Southwest (5) ❍ Other (6)
The following questions refer to your knowledge of kratom:
Q9 Have you heard of kratom before?
❍ Yes (1) ❍ No (2)
Q10 If you have heard of kratom before, from where or whom did you hear about it?
❍ Pharmacy or medical school curriculum (1) ❍ Continuing education article/program (2) ❍ Pharmacist (3) ❍ Scientific/medical literature (4) ❍ Lay-press article or news (5)
Q11 How would you rate your overall knowledge of kratom?
❍ Not at all knowledgeable (1) ❍ Not very knowledgeable (2) ❍ Neutral (3) ❍ Knowledgeable (4) ❍ Very knowledgeable (5)
Q12 How confident are you that you could list common reasons why people take kratom?
❍ Not at all confident (1) ❍ Not very confident (2) ❍ Neutral (3) ❍ Confident (4) ❍ Very confident (5)
Q13 How confident are you that you could describe the clinical effects of kratom?
❍ Not at all confident (1) ❍ Not very confident (2) ❍ Neutral (3) ❍ Confident (4) ❍ Very confident (5)
Q14 How confident are you that you could describe the legal issues surrounding kratom?
❍ Not at all confident (1) ❍ Not very confident (2) ❍ Neutral (3) ❍ Confident (4) ❍ Very confident (5)
Q15 Has a patient or acquaintance ever asked you about kratom?
❍ Yes (1) ❍ No (2)
Q16 Have you ever read an article about kratom in a scientific or medical journal?
❍ Yes (1) ❍ No (2)
Q17 Have you ever read an article in the lay press about kratom?
❍ Yes (1) ❍ No (2)
Q18 Have you ever heard of mitragynine or 7-hydroxymitragynine?
❍ Yes (1) ❍ No (2)
Q19 Are you aware of how kratom is typically ingested (i.e., swallowed, inhaled, injected, etc.)?
❍ Yes (1) ❍ No (2)
